# Redistribution of peripheral blood immune cell subsets and remodeling of intercellular communication in rare Behçet's disease: a single-cell transcriptomics study

**DOI:** 10.3389/fmed.2026.1908366

**Published:** 2026-08-04

**Authors:** Yanfei Yang, Yuexin Chen

**Affiliations:** Department of Vascular Surgery, Peking Union Medical College Hospital, Chinese Academy of Medical Sciences and Peking Union Medical College, Beijing, China

**Keywords:** Behçet's disease, intercellular communication differential analysis, LASSO diagnostic biomarker screening, monocyte subsets, peripheral blood immune cells, peripheral blood single-cell RNA sequencing

## Abstract

**Background:**

Behçet's disease (BD) is a rare chronic systemic vasculitis of unknown etiology. The heterogeneity of peripheral blood immune cells and the dysregulation of intercellular communication in BD remain insufficiently characterized. The present study employs single-cell transcriptomics to comprehensively delineate the peripheral blood immune cell atlas of BD, identify candidate diagnostic biomarkers, and dissect the remodeling patterns of intercellular communication networks.

**Methods:**

Single-cell RNA sequencing data of peripheral blood mononuclear cells (PBMCs) from 8 BD patients and 8 healthy controls (HCs) (GSE198616) were analyzed using Seurat v5, clusterProfiler, LASSO regression, and CellChat v1.6. Differential expression analysis at both single-cell and pseudobulk levels, GSEA, and ssGSEA were performed, with external validation carried out using two independent transcriptomic datasets (GSE61399 and GSE205867).

**Results:**

Nine major immune cell types were identified, and monocytes were further subdivided into three subsets. BD patients exhibited subset-specific monocyte redistribution at the group level: CD14^+^ classical monocytes showed a significantly higher proportion (8.54% vs. 1.51%, *p* < 0.001), whereas FCGR3A^+^ non-classical monocytes were significantly reduced (3.04% vs. 7.23%, *p* < 0.001), accompanied by elevated proportions of CD8^+^ T cells, CD4^+^ T cells, plasma cells, and proliferating cells. Differential expression analysis identified 86 upregulated and 84 downregulated genes, enriched in interferon and inflammatory pathways. LASSO regression selected nine feature genes; IFI44L demonstrated moderate diagnostic potential (AUC = 0.734, 95% CI: 0.449–1, *p* = 0.1304). Pseudobulk validation at the individual level confirmed consistent upregulation of the core genes IFI44L, GBP1, and FCGR1A in BD. Intercellular communication analysis revealed attenuated costimulatory signaling (CD40, ICAM) in BD, whereas the ANNEXIN pathway was significantly enhanced in CD8^+^ T cells, plasma cells, and proliferating cells, and PECAM1 was increased in plasma cells and proliferating cells but reduced in monocytes. External validation confirmed consistent upregulation of LASSO-selected core genes in CD14^+^ monocytes, along with robust activation of type I interferon signaling in both CD14^+^ monocytes (NES = +3.04, FDR < 0.001) and CD4^+^ T cells (NES = +2.96, FDR < 0.001). Notably, monocytes displayed overall communication suppression despite strong transcriptional activation of interferon signaling, suggesting a subtype-specific regulatory state of being transcriptionally active yet communication-suppressed.

**Conclusion:**

This study delineates the peripheral blood immune landscape of BD, revealing subset-specific monocyte redistribution and subtype-specific remodeling of intercellular communication. These findings generate new hypotheses for diagnostic biomarker development and communication-targeted therapeutic strategies in BD, warranting further experimental validation in well-phenotyped cohorts.

## 1 Introduction

Behçet's disease (BD) is a multi-organ systemic vasculitic disorder recognized as a rare disease (1, 2). Its clinical manifestations are highly heterogeneous and can be life-threatening (3, 4). The pathogenesis of BD is exceedingly complex, exhibiting features of both autoimmunity and autoinflammation (5, 6). BD can involve the oral mucosa, skin, eyes, blood vessels, and central nervous system, and is characterized by recurrent episodes of relapse and remission (7, 8). Although aberrant activation of both innate and adaptive immunity is thought to contribute to BD pathogenesis, the fine compositional subsets of peripheral blood immune cells, their transcriptomic signatures, and the systematic alterations in intercellular communication networks remain incompletely elucidated (9–12). In particular, whether specific immune cell subsets exhibit a “quantity–function” imbalance, and how such an imbalance drives persistent inflammation through distinct signaling pathways, represents a notable gap in current knowledge.

To address this gap, we leveraged single-cell RNA sequencing data from the public dataset GSE198616 (eight BD patients and eight healthy controls) to systematically profile the peripheral blood immune cell landscape in BD. We screened candidate diagnostic genes and characterized the remodeling of intercellular communication networks between BD and healthy controls. By integrating differential expression analysis, LASSO-based diagnostic modeling, and CellChat-mediated intercellular communication comparison, we focused on communication changes across seven candidate inflammatory/metabolic pathways (CCL, CysLTs, RESISTIN, ANNEXIN, PECAM1, Cholesterol, and BAFF) within five key immune cell types (CD8^+^ T cells, CD4^+^ T cells, plasma cells, proliferating cells, and monocytes, with CD14^+^ classical monocytes predominating).

The potential roles of these pathways in BD pathogenesis are supported by existing literature. For instance, CCL3 exacerbates symptoms in a BD mouse model (13). CysLTs (cysteinyl leukotrienes) are potent inflammatory mediators that induce vascular smooth muscle contraction and increase vascular permeability (14). Resistin, a pro-inflammatory adipokine, is elevated in the serum of BD patients and positively correlates with disease activity, suggesting its utility as a biomarker of disease activity (15). Annexin A2 has been identified as an autoantigen in BD vascular endothelial cells, and anti-annexin V autoantibodies are associated with an increased risk of thrombotic complications (16, 17). PECAM-1 (CD31) is an important adhesion molecule involved in angiogenesis and leukocyte transendothelial migration; its expression is upregulated in BD skin lesions, contributing to vascular inflammation and modulating B cell development (18). Regarding lipid metabolism, studies have reported significantly elevated HDL levels and potentially reduced total cholesterol (TC) in BD patients (19). BAFF is markedly elevated in the serum of patients with active BD and positively correlates with skin lesion severity (20). Collectively, this evidence provides a solid rationale for focusing on these seven pathways in the present study.

Building on this foundation, we propose that the inflammatory cascade in BD constitutes an interconnected pathological sequence rather than a single event. CCL-driven chemotaxis and cholesterol metabolic dysregulation may initiate vascular wall inflammation; resistin and CysLTs subsequently amplify this response and propel disease progression. As inflammation advances, annexin family members become targets of autoimmune attack, and PECAM-1 regulated adhesion and migration alterations lead to structural damage of the vessel wall. BAFF-driven humoral abnormalities may further establish immune memory, offering an explanation for the recurrent, chronic course of BD. Taken together, these pathways delineate a continuum from initiation to perpetuation.

In summary, by integrating single-cell transcriptomics with intercellular communication analysis, this study characterizes how the peripheral blood immune landscape is remodeled in BD and where intercellular signaling goes awry. We anticipate that these results will shed light on the mechanisms underlying persistent inflammation in BD, reveal the decoupling phenomenon of “transcriptionally active yet communication-suppressed” immune cells, and point toward candidate biomarkers and communication-based intervention targets worthy of further investigation.

## 2 Methods

### 2.1 Data acquisition and pre-processing

The GSE198616 dataset was downloaded from the GEO database (https://www.ncbi.nlm.nih.gov/geo/query/acc.cgi?acc=GSE198616), comprising peripheral blood mononuclear cells (PBMCs) and CD14^+^ monocytes from 8 BD patients and 8 healthy controls (HCs), sequenced on the 10X Genomics platform. The Seurat v5 package (R 4.3) (Satija Lab, New York Genome Center, New York, USA) was used to read the three files (barcodes.tsv.gz, features.tsv.gz, matrix.mtx.gz) for each sample, creating an independent Seurat object per sample. Quality-control filtering criteria were as follows: genes detected per cell (nFeature_RNA) between 200 and 2,500, and mitochondrial gene fraction (percent.mt) < 20%.

### 2.2 Seurat integration workflow (batch effect correction)

Each sample's Seurat object was normalized using LogNormalize, and highly variable genes were identified (nfeatures = 2,000). The FindIntegrationAnchors function (dims = 1:30) was employed to identify cross-sample anchors, after which all samples were integrated via the IntegrateData function to generate an integrated data matrix. The integrated data served as the basis for subsequent dimensionality reduction, clustering, and differential analyses, thereby mitigating batch effects.

### 2.3 Dimensionality reduction, clustering, and cell type annotation

Monocytes formed three distinct clusters in the UMAP projection, representing CD14^+^ classical monocytes (cluster 7), FCGR3A^+^ non-classical monocytes (cluster 9), and FCN1^+^ myeloid progenitors (cluster 11). These subsets were merged into a unified “Monocyte” annotation for downstream analyses. Clusters 18–23, which lacked definitive lineage-specific markers, were classified as “unknown cells” and excluded from downstream analyses because they accounted for less than 3% of total cells. Clusters 13–17, although displaying ambiguous marker expression, were retained as an “unknown” category during visualization but were not included in cell proportion comparisons. Principal component analysis (PCA) was performed on the integrated data, with the first 50 principal components retained. Unsupervised clustering was carried out using FindNeighbors (dims = 1:30) and FindClusters (resolution = 0.5). UMAP was used for visualization. Each cluster was manually annotated based on known cell marker genes (CD3D, CD4, CD8A, MS4A1, CD14, NKG7, FCGR3A, etc.). To quantify subset-specific proportional differences between BD and HC, we calculated the percentage of each monocyte subset relative to total PBMCs per sample and compared the BD and HC groups using a two-sided Wilcoxon rank-sum test. *P* < 0.05 was considered statistically significant.

### 2.4 Differential expression analysis

With disease status as the grouping variable (BD vs. HC), differentially expressed genes (DEGs) were identified using the FindMarkers function (Wilcoxon rank-sum test). Thresholds were set at |log2FC| > 0.25 and adjusted *p*-value (Bonferroni) < 0.05. For visualization, the significance threshold was raised to |log2FC| > 0.5.

We acknowledge that differential expression analysis at the single-cell level may suffer from pseudoreplication, as cells derived from the same individual do not constitute independent biological replicates. Consequently, the DEG results should be regarded as a discovery screen rather than definitive statistical inference. Importantly, our key findings were subsequently validated using two independent bulk transcriptomic datasets (GSE61399 and GSE205867), in which each sample represents an independent biological replicate.

To address pseudoreplication concerns, we performed pseudobulk differential expression analysis by aggregating the mean expression of each gene per donor using the AggregateExpression function in Seurat, followed by DESeq2 analysis with disease status as the grouping variable.

### 2.5 Functional enrichment analysis

GO (BP, CC, MF) and KEGG pathway enrichment analyses were performed separately for upregulated and downregulated genes using the clusterProfiler package. The significance threshold was p.adjust < 0.05 (Benjamini–Hochberg method). Results were visualized using barplot, dotplot, and cnetplot. GO Cellular Component (CC) terms were excluded from the final presentation, as the enriched CC terms were predominantly general categories (e.g., membrane, cytoplasm) that failed to provide disease-relevant biological insights beyond the BP and MF analyses.

### 2.6 Diagnostic marker screening and model performance evaluation

Based on all DEGs (p_adj < 0.05, |log2FC| > 0.25), LASSO regression (10-fold cross-validation, binomial distribution, alpha = 1) was performed using the glmnet package to select genes with non-zero coefficients. The mean expression of these genes per sample (pseudo-bulk expression matrix) was computed to construct a multivariable logistic regression model. Receiver operating characteristic (ROC) curves were plotted for individual genes and the combined model, and the area under the curve (AUC), sensitivity, specificity, and optimal cutoff (Youden index) were calculated.

### 2.7 Intercellular communication analysis

The CellChat package (v1.6) (GitHub repository (https://github.com/sqjin/CellChat) was used to construct intercellular communication networks separately for the BD and HC groups. The ligand–receptor database CellChatDB.human was employed, communication probabilities were computed (computeCommunProb), and interactions with probability < 0.05 were filtered out. Total communication strength (netVisual_heatmap) and signaling pathway differences (netVisual_bubble) were compared between the two groups.

### 2.8 CellChat network construction and comparison

#### 2.8.1 Data source and pre-processing

Based on the ligand–receptor interaction lists output by the CellChat package (from the BD and HC groups separately), we obtained all detected intercellular communication events in each sample, including sender cells, receiver cells, associated signaling pathway names, and communication probabilities (prob). The two groups were then merged with group labels (BD or HC) for subsequent differential analysis.

#### 2.8.2 Overall communication comparison by cell type

Based on the UMAP clustering results, we identified cell types that were more abundant in the BD group than in the HC group: CD8^+^ T cells, CD4^+^ T cells, plasma cells, proliferating cells, and monocytes (predominantly CD14^+^ classical monocytes). To investigate changes in their communication activity under disease conditions, we extracted all communication events involving each of these cell types as either sender or receiver. For each cell type, the distributional differences in communication probabilities between the BD and HC groups were compared using a two-sided Wilcoxon rank-sum test, with box plots displaying the median, interquartile range, and overall distribution for each group. Significance levels were automatically annotated on the box plots (^*^p < 0.05, ^**^p < 0.01, ^***^p < 0.001; ns indicates no significant difference). This analysis was designed to assess, at an overall level, whether the communication activity of each immune cell type was altered in BD. To further dissect the directionality of monocyte communication, we separately extracted communication events in which monocytes served as senders (source = = “Monocyte”) and as receivers (target = = “Monocyte”), comparing communication probabilities between the BD and HC groups to evaluate functional changes in both signal output and input directions.

#### 2.8.3 Communication differential analysis of specific signaling pathways

Based on preliminary differential pathway screening (obtained via the rankNet function), we selected seven signaling pathways with substantial information flow differences between the BD and HC groups and potential biological relevance: CCL, CysLTs, RESISTIN, ANNEXIN, PECAM1, Cholesterol, and BAFF. For each of the aforementioned five cell types, we extracted communication events involving that cell (as either sender or receiver) within each pathway. Subsequently, for each “cell type–pathway” combination, communication probabilities were compared between the BD and HC groups, again using the Wilcoxon rank-sum test and box plots with significance markers for visualization. This analysis aimed to reveal through which inflammatory or metabolism-related pathways specific immune cell subsets undergo communication remodeling in BD.

### 2.9 External validation using independent transcriptomic datasets

To validate the generalizability of our findings, we utilized two independent public datasets—GSE61399 (Affymetrix microarray, purified CD14^+^ monocytes and CD4^+^ T cells) and GSE205867 (Illumina RNA-seq, peripheral blood neutrophils)—for external validation of the expression patterns of the nine LASSO-selected core genes and the activation of interferon signaling pathways. For GSE61399, differential expression analysis was performed using the limma package; genes with FDR < 0.05 and |log2FC| > 0.585 were considered significant. For GSE205867, DESeq2 was used to process raw counts; genes with padj < 0.05 and |log2FC| > 0.5 were considered significant. Gene set enrichment analysis (GSEA) was conducted using the clusterProfiler package based on MSigDB Hallmark and KEGG gene sets. Single-sample GSEA (ssGSEA) was performed using the GSVA package to quantify pathway activity scores for CCL, PECAM1, ANNEXIN, and interferon response pathways. All statistical analyses were performed in R 4.3.

### 2.10 Visualization and statistics

Figures were generated using ggplot2 (Hadley Wickham, Rice University, Houston, USA), pheatmap, VennDiagram (Hanbo Chen and Paul C. Boutros, Ontario Institute for Cancer Research, Toronto, Canada), and other packages. All statistical analyses were performed in R 4.3, with *p* < 0.05 considered statistically significant.

## 3 Results

### 3.1 Single-cell atlas of peripheral blood in BD patients, immune landscape remodeling, and identification of differentially expressed genes

**Figure 1:**
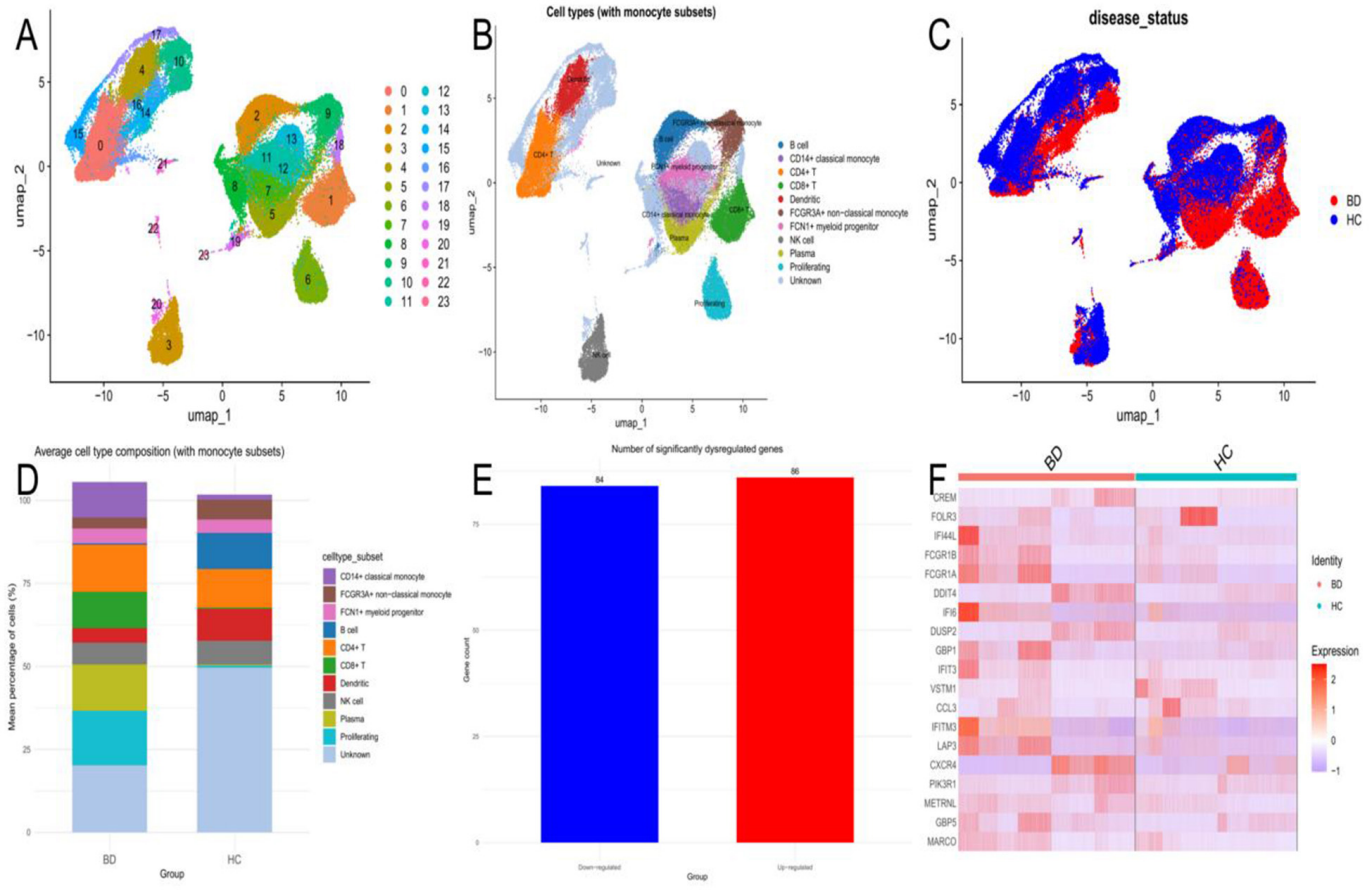
Single-cell transcriptomic landscape and immune atlas of peripheral blood in Behçet's disease (BD). **(A, B)** UMAP of all profiled cells colored by nine annotated cell types. **(C)** UMAP colored by disease status (BD vs. HC). **(D)** Cell type proportions in BD and HC. **(E)** Numbers of upregulated (86) and downregulated (84) DEGs (|log2FC| > 0.5, p_adj < 0.05). **(F)** Heatmap of representative DEGs (e.g., IFI44L, FCGR1A, IFI6, IFIT3, IFITM3, LAP3). BD, Behçet's disease; HC, healthy control; UMAP, uniform manifold approximation and projection; DEG, differentially expressed gene; FC, fold change; p_adj, adjusted *p*-value.

Following data processing, UMAP dimensionality reduction plots were generated. Each color represents one cell type, with colors indicating nine manually annotated cell types (Figure 1B: UMAP colored by cell type; Figure 1A: UMAP colored by cell type with cluster labels). Figure 1C shows the UMAP colored by disease status. Unknown cells (clusters 18–23) were excluded from cell proportion analyses due to ambiguous marker expression, as they accounted for less than 3% of total cells. Figure 1D displays the relative percentages of each cell type in the BD and HC groups. Compared with the HC group, the BD group showed significantly elevated proportions of CD8^+^ T cells, CD4^+^ T cells, plasma cells, proliferating cells, and CD14^+^ classical monocytes, a mildly increased proportion of FCN1^+^ myeloid progenitors, and decreased proportions of FCGR3A^+^ non-classical monocytes, B cells, dendritic cells, and NK cells. Individual-level UMAP projections (Supplementary Figure S3), colored by cluster identity, confirmed that the clustering patterns were broadly consistent across individuals within each group, with no single donor driving the overall differences. The cluster-specific cell type annotations are provided in Figure 1B.

Subsequently, differential gene expression analysis was performed. At thresholds of |log2FC| > 0.5 and p_adj < 0.05, a total of 86 upregulated genes (red) and 84 downregulated genes (blue) were identified (Figure 1E). Figure 1F presents a heatmap of differentially expressed genes, highlighting genes such as IFI44L, FCGR1A, IFI6, IFIT3, IFITM3, and LAP3.

Clustering analysis of monocyte subsets revealed distinct abundance patterns for the three subsets between BD and HC at the group level. CD14^+^ classical monocytes (cluster 7) showed a significantly higher proportion in BD (8.54% vs. 1.51% of total PBMCs, *p* < 0.001), whereas FCGR3A^+^ non-classical monocytes (cluster 9) were markedly reduced (3.04% vs. 7.23%, *p* < 0.001). FCN1^+^ myeloid progenitors (cluster 11) showed a modest, non-significant increase in BD (4.76% vs. 4.19%, ns). Per-subject scatter plots (Supplementary Figure S1B) revealed considerable inter-individual variability within each group, with directional trends higher CD14^+^ classical monocytes and lower FCGR3A^+^ non-classical monocytes in BD, observed in the majority of BD patients. These findings indicate that the changes in monocyte composition in BD are subset-specific rather than a uniform reduction across all monocytes. Supplementary Figure S1A presents the group-level proportions, and Supplementary Figure S1B shows the individual-level distributions. While the group-level differences were statistically significant, the individual-level scatter plots highlight the heterogeneity among patients within each group, underscoring the importance of interpreting these findings in the context of the limited sample size.

### 3.2 GO and KEGG enrichment analysis

**Figure 2:**
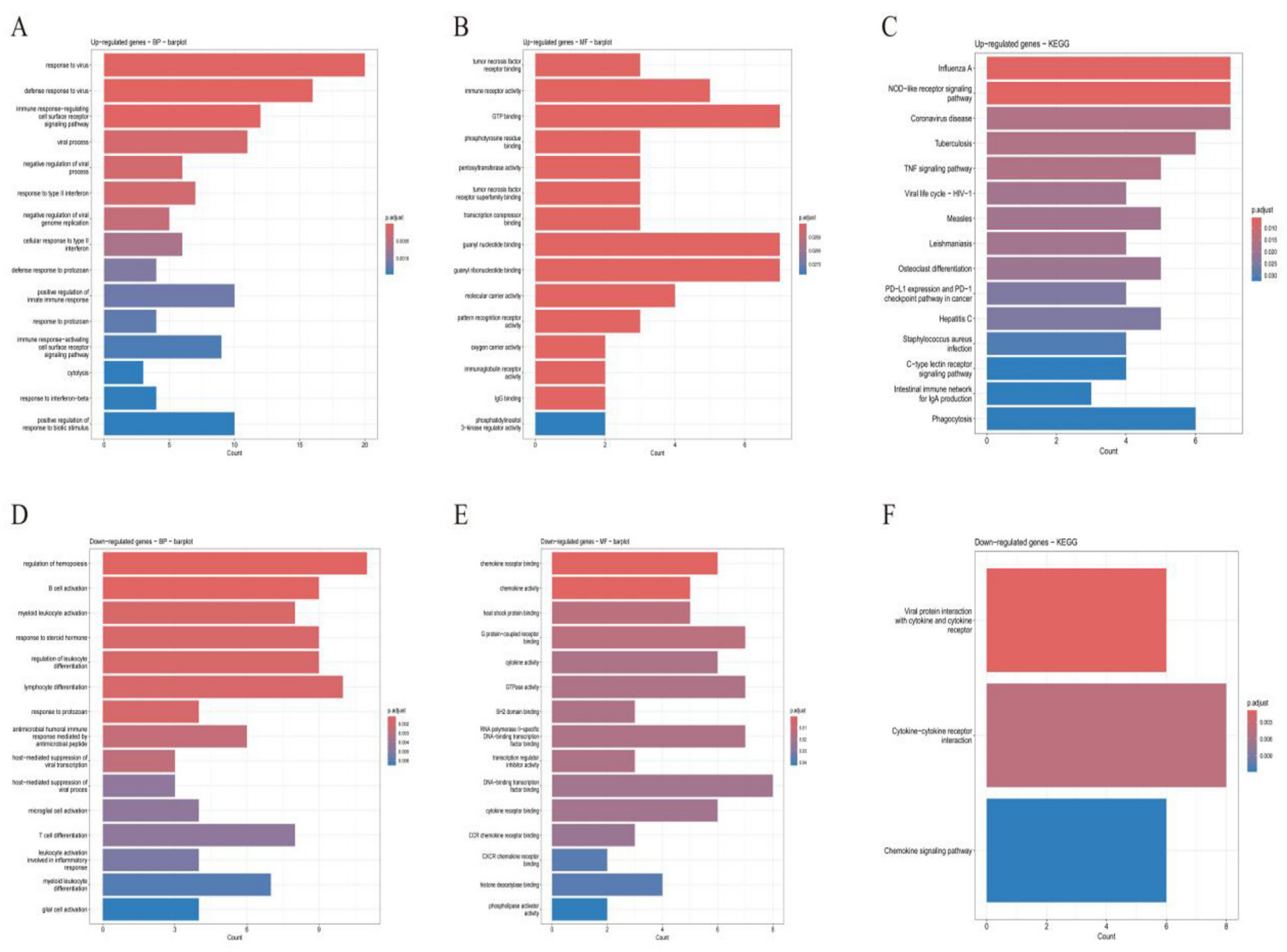
Functional enrichment analysis of differentially expressed genes in BD. **(A)** GO Biological Process (BP) enrichment analysis of upregulated genes. **(B)** GO Molecular Function (MF) enrichment analysis of upregulated genes. **(C)** KEGG pathway enrichment analysis of upregulated genes. **(D)** GO BP enrichment analysis of downregulated genes. **(E)** GO MF enrichment analysis of downregulated genes. **(F)** KEGG pathway enrichment analysis of downregulated genes.GO Cellular Component (CC) terms were not included as they did not yield disease-relevant biological insights. Significance threshold: p.adjust < 0.05 (Benjamini–Hochberg).

GO Biological Process (BP) and Molecular Function (MF) enrichment analyses of upregulated genes are shown in Figures 2A, B, respectively; KEGG pathway analysis of upregulated genes is presented in Figure 2C. GO BP and MF analyses of downregulated genes are shown in Figures 2D, E, respectively; KEGG analysis of downregulated genes is shown in Figure 2F. GO Cellular Component (CC) terms were excluded, as they failed to provide disease-relevant biological insights. GO-BP terms enriched among upregulated genes included “interferon gamma response,” “inflammatory response,” and “neutrophil activation.” Significantly enriched KEGG pathways included the JAK-STAT signaling pathway, NOD-like receptor pathway, and TNF signaling pathway.

### 3.3 ROC analysis of the top 20 differentially expressed genes and assessment of disease predictive capacity

**Figure 3:**
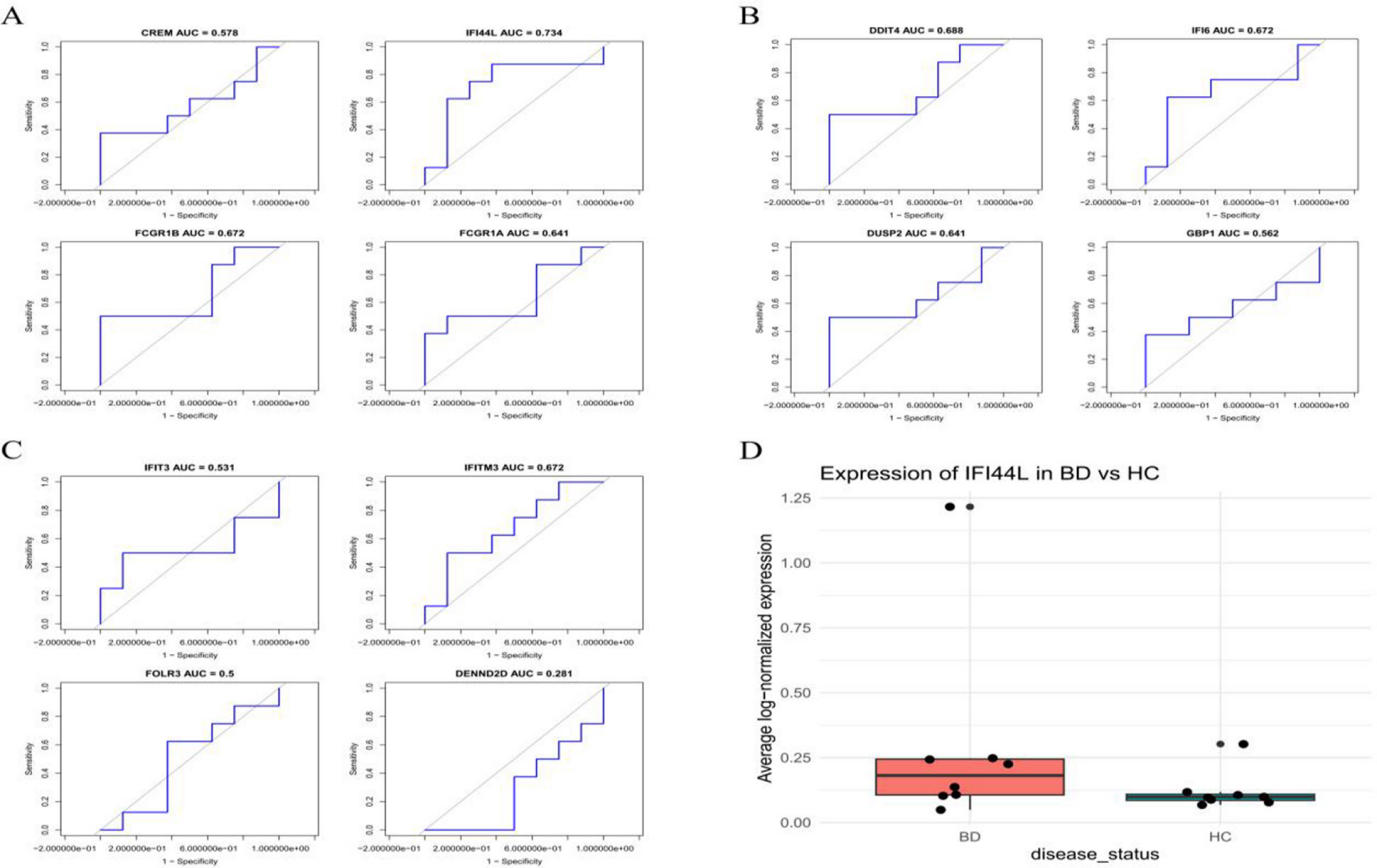
Assessment of disease prediction ability using top differentially expressed genes in BD. **(A–C)** Receiver operating characteristic (ROC) curves of the top 12 differentially expressed genes, demonstrating their ability to distinguish BD from HC. **(D)** Boxplot showing expression levels of IFI44L in BD vs. HC groups (AUC = 0.734, 95% CI: 0.449–1; Wilcoxon rank-sum test, *p* = 0.1304). Although the AUC suggested moderate discriminative ability, statistical significance was not reached, likely due to the limited sample size.

ROC curves were plotted for the top 20 differentially expressed genes, with the first 12 genes visualized in Figures 3A–C. A total of 10 genes with AUC > 0.5 were identified, including CREM, IFI44L, DDIT4, IFI6, FCGR1B, FCGR1A, DUSP2, GBP1, IFIT3, and IFITM3. IFI44L expression tended to be higher in BD patients than in HCs (median: BD = 0.1812, HC = 0.0981; fold change = 1.85; Wilcoxon rank-sum test, *p* = 0.1304; AUC = 0.734, 95% CI: 0.449–1; Figure 3D). Although the AUC suggested moderate discriminative capacity, the difference did not reach statistical significance, likely due to the limited sample size (*n* = 8 per group). IFI44L was highlighted among the LASSO-selected genes for three reasons: (i) it carried the highest absolute coefficient in the lambda.min model; (ii) it exhibited the highest individual AUC (0.734) among the nine feature genes; and (iii) it is a well-established interferon-stimulated gene, mechanistically linking our diagnostic marker to the interferon signaling pathway, which was the most significantly enriched pathway in our GO analysis. To assess whether the observed trend was driven by outlier samples, we performed a leave-one-out analysis, removing one BD sample at a time. The log2FC remained positive across all iterations (range: 0.41–1.45; Supplementary Table S3), confirming that the upregulation trend was not attributable to any single sample.

### 3.4 LASSO regression-based diagnostic marker screening

**Figure 4:**
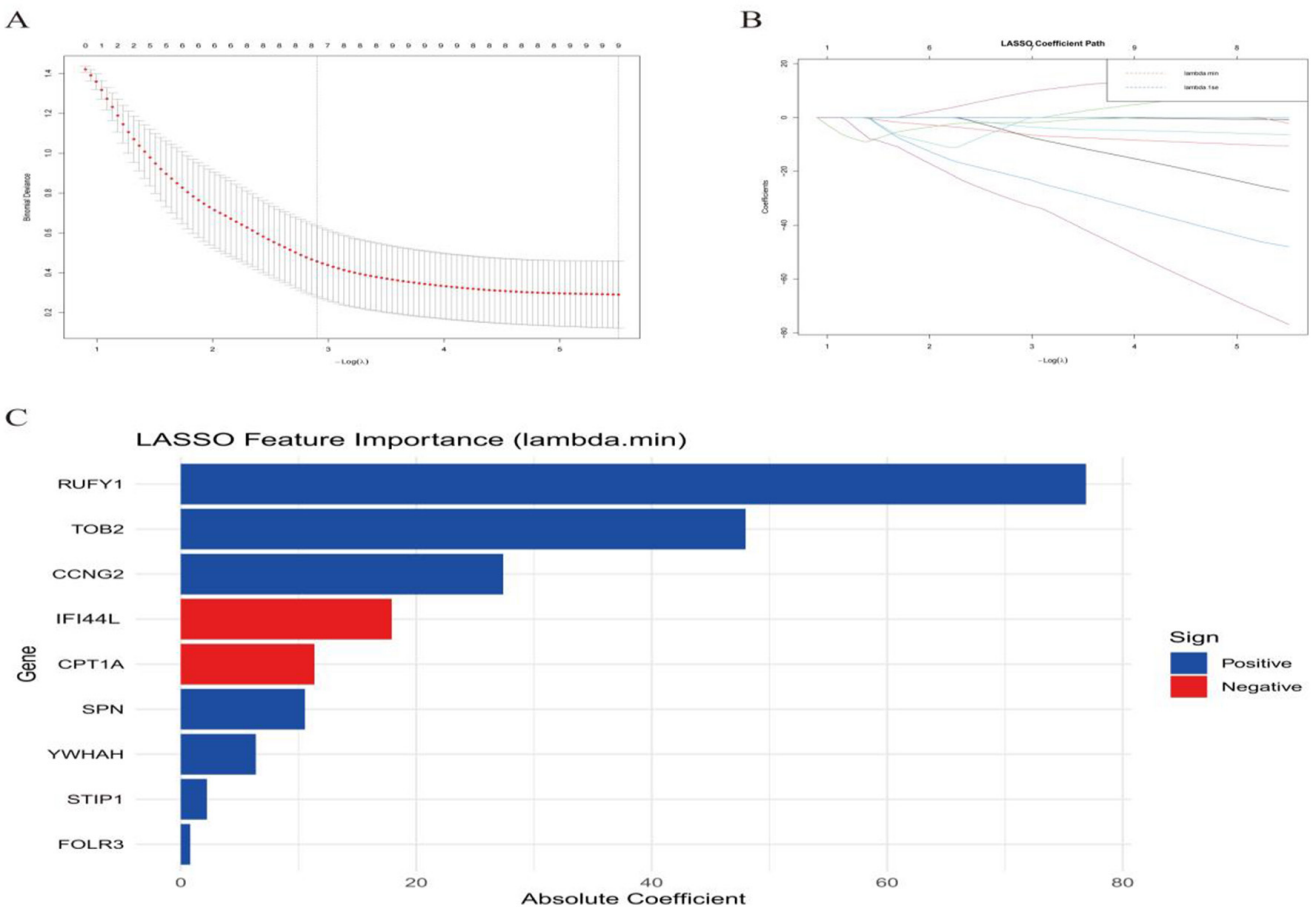
LASSO regression for diagnostic biomarker screening. **(A)** Cross-validation error curve for LASSO regression. The y-axis shows binomial deviance, and the x-axis shows log(λ). The red vertical dashed line indicates λ.min (minimum error), and the blue vertical dashed line indicates λ0.1se (most parsimonious model). **(B)** Coefficient path plot showing shrinkage of gene coefficients as λ increases. Nine genes retained non-zero coefficients at λ.min. **(C)** Bar plot of absolute coefficient values for the nine selected genes at λ.min. RUFY1, TOB2, CCNG2, and IFI44L showed the highest contributions.

The LASSO cross-validation error curve was generated, with binomial deviance on the y-axis and log(lambda) on the x-axis. The red vertical dashed line corresponds to lambda.min (minimum error), and the blue dashed line corresponds to lambda.1se (most parsimonious model) (Figure 4A). The coefficient path plot illustrates how each gene's coefficient shrinks with increasing lambda, ultimately yielding nine genes with non-zero coefficients at lambda.min (Figure 4B). The feature importance bar chart displays the absolute coefficients of the nine genes in the lambda.min model, with RUFY1, TOB2, CCNG2, and IFI44L contributing the most (Figure 4C).

To address potential pseudoreplication concerns, we performed an additional pseudobulk differential expression analysis by aggregating cells per donor (DESeq2, *n* = 8 BD vs. 8 HC). The LASSO-selected core genes showed consistent upregulation trends in BD: IFI44L (log2FC = +1.55, *p* = 0.004), FCGR1A (log2FC = +1.45, *p* = 0.047), and GBP1 (log2FC = +1.29, *p* = 0.010). However, statistical significance was attenuated due to the limited sample size, with only TOB2 reaching padj < 0.05 (log2FC = −0.82, padj = 0.034). These results confirm the directional consistency of our key findings at the individual level and are presented in Supplementary Table S4.

### 3.5 Intercellular communication analysis

**Figure 5:**
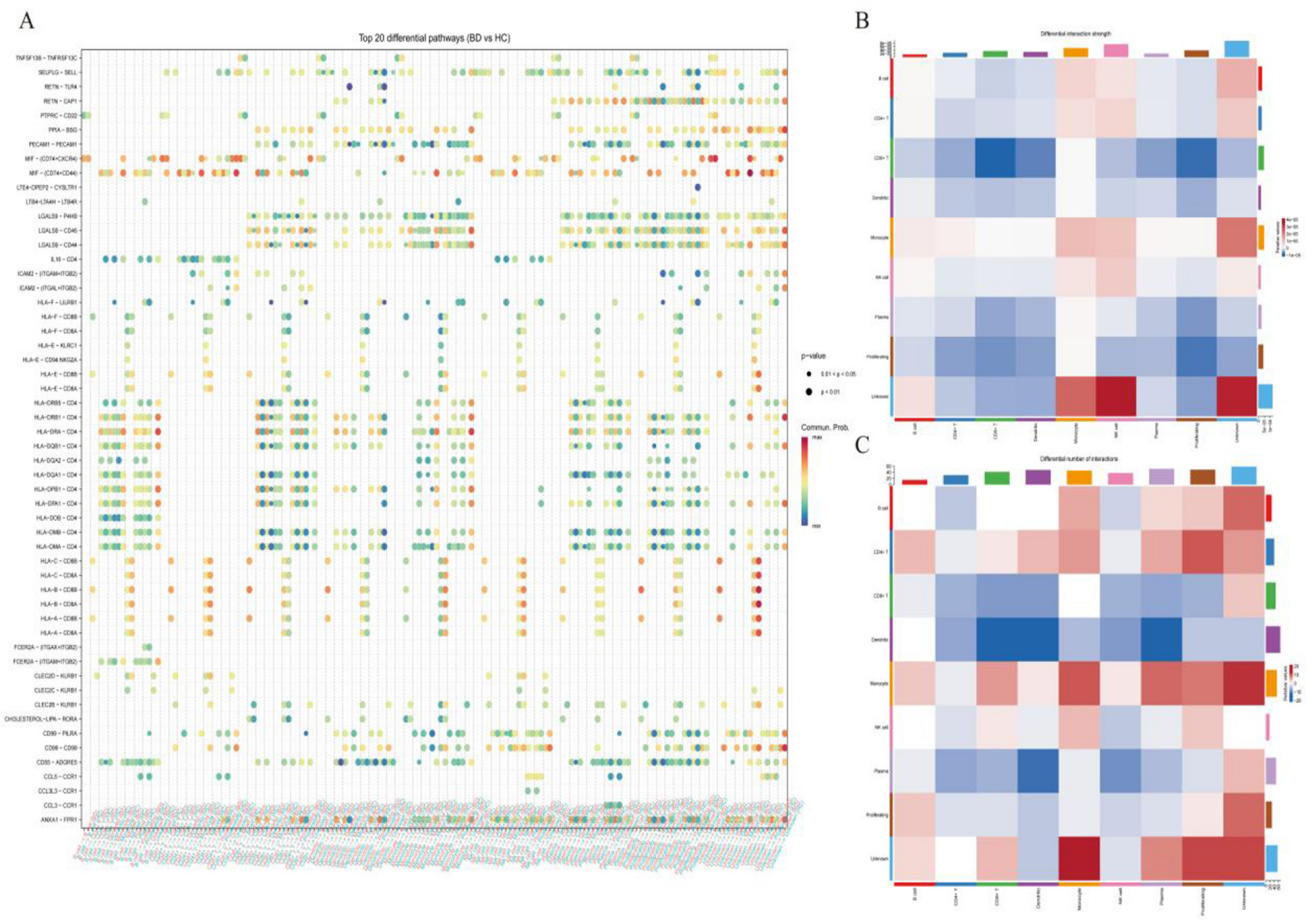
Alterations in cell-cell communication networks between BD and HC. **(A)** Comparison of information flow intensity for the top 20 differentially regulated signaling pathways between BD and HC. Pathways stronger in HC include CD40, APP, SEMA4, ICAM, CD23, LCK, IL16, CLEC, MHC-I, SELPLG, etc. Pathways stronger in BD include CCL, CysLTs, RESISTIN, ANNEXIN, PECAM1, Cholesterol, and BAFF. **(B, C)** Heatmaps showing the number of cell-cell communication interactions in BD **(B)** and HC **(C)**. Redder color indicates a greater number of communication pairs.

Based on the merged CellChat object, information flow intensities of signaling pathways were compared between the BD and HC groups, with the top 20 most differential pathways shown in Figure 5A (detailed statistical information in Supplementary Table S1: top20_differential_pathways). Heatmaps of communication counts between cell types in the BD and HC groups are shown in Figures 5B, C, where redder colors indicate more communication pairs. Pathways including CD40, APP, SEMA4, ICAM, CD23, LCK, IL16, CLEC, MHC-I, and SELPLG were stronger in the HC group (i.e., relatively attenuated or absent in BD). Pathways such as CCL, CysLTs, RESISTIN, ANNEXIN, PECAM1, Cholesterol, and BAFF were stronger in the BD group (among which CysLTs and RESISTIN showed the most pronounced differences, with deltas of 0.0267 and 0.0107, respectively; all other pathway differences were < 0.01; see Supplementary Table S2: top20_BD_contribution). We further filtered for pathways with greater contribution to the BD group relative to the HC group and ultimately identified CCL, RESISTIN, ANNEXIN, and PECAM1 as the most differential (Supplementary Figures S2A–D). For the CysLTs, Cholesterol, and BAFF pathways, insufficient data precluded box plot visualization (data not shown).

### 3.6 Overall communication comparison analysis by cell type

**Figure 6:**
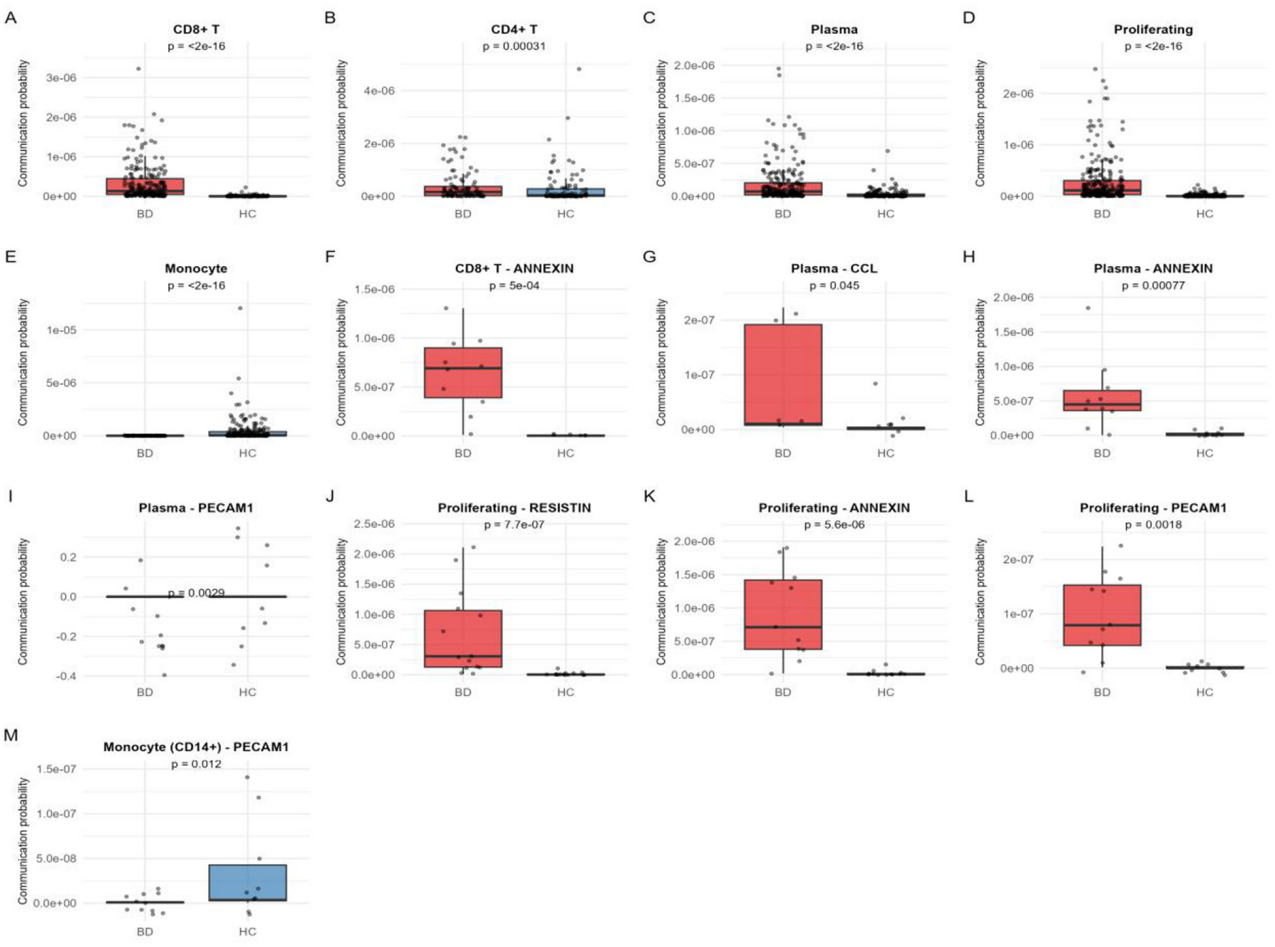
Cell-type-specific communication differences in BD. **(A–E)** Overall communication probability of CD8+ T cells **(A)**, CD4+ T cells **(B)**, plasma cells **(C)**, proliferating cells **(D)**, and monocytes [**(E)**; merged ‘Monocyte' population, primarily representing CD14+ classical monocytes] in BD vs. HC. All four immune subsets **(A–D)** showed significantly elevated communication in BD (*p* < 0.001), whereas monocytes **(E)** exhibited significantly reduced communication (*p* < 0.001). **(F–M)** Cell-type-specific communication differences for seven inflammatory/metabolic pathways. Significant differences were observed for the following combinations: ANNEXIN in CD8^+^ T cells **(F)**; CCL in plasma cells **(G)**; ANNEXIN in plasma cells **(H)** and proliferating cells **(K)**; PECAM1 in plasma cells **(I)** and proliferating cells **(L)**; RESISTIN in proliferating cells **(J)**; and PECAM1 in monocytes **(M)**. Notably, PECAM1 was significantly decreased in monocytes **(M)**, while all other significant combinations showed increased communication in BD. Pathways CysLTs, Cholesterol, and BAFF were excluded due to sparse data. Statistical significance: *p < 0.05, **p < 0.01, ***p < 0.001 (Wilcoxon rank-sum test). BD, Behçet's disease; HC, healthy control.

Our initial UMAP analysis revealed that, compared with the HC group, the BD group exhibited significantly elevated proportions of CD8^+^ T cells, CD4^+^ T cells, plasma cells, proliferating cells, and monocytes (predominantly CD14^+^ classical monocytes), a mildly increased proportion of FCN1^+^ myeloid progenitors, and significantly decreased proportions of FCGR3A^+^ non-classical monocytes, B cells, dendritic cells, and NK cells. Based on these differences, we further focused on exploring communication activity changes in CD8^+^ T cells, CD4^+^ T cells, proliferating cells, plasma cells, and monocytes (predominantly CD14^+^ classical monocytes) under disease conditions. For each cell type, the distributional differences in communication probabilities between the BD and HC groups were compared. The results showed that overall communication probabilities for CD8^+^ T cells (Figure 6A), CD4^+^ T cells (Figure 6B), plasma cells (Figure 6C), and proliferating cells (Figure 6D) were all significantly higher in the BD group than in the HC group (*p* < 0.001), whereas the overall communication probability for monocytes (Figure 6E) was significantly lower in the BD group (*p* < 0.001). Notably, despite the significant proportional increase of CD14^+^ classical monocytes in BD, the merged “Monocyte” population exhibited a significantly reduced overall communication probability. This suggests that non-classical monocytes may be the principal contributors to monocyte communication, and their selective loss is sufficient to dominate the overall downregulation of communication.

### 3.7 Comparative analysis of pathway-specific communication differences by cell type

**Figure 7:**
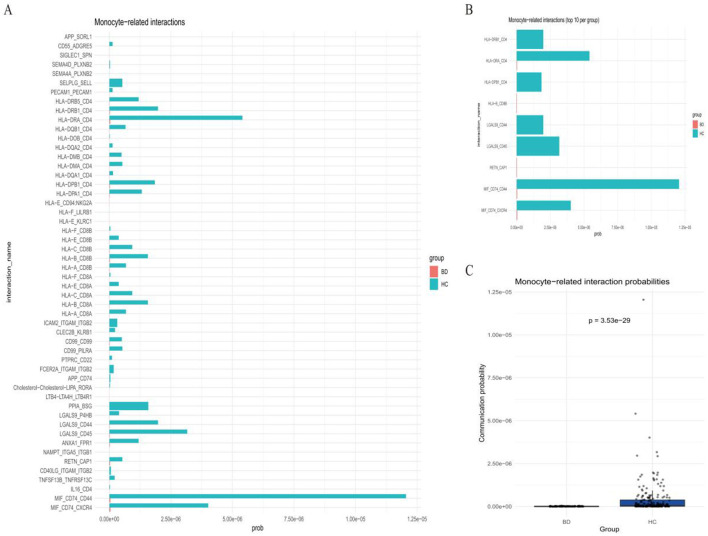
Reduced monocyte-related communication in BD. **(A–C)** Monocyte-focused cell-cell communication network analysis, showing that the interaction probability between monocytes and other cell types is significantly reduced in BD compared with HC. Each subpanel illustrates a different aspect of monocyte-centric communication, all indicating weakened outgoing/incoming signals of monocytes in BD.

Based on the preliminary differential pathway screening (Figure 5A), we further evaluated communication probability differences across seven inflammatory/metabolic pathways (CCL, CysLTs, RESISTIN, ANNEXIN, PECAM1, Cholesterol, and BAFF) within five target immune cell types (CD8^+^ T cells, CD4^+^ T cells, plasma cells, proliferating cells, and monocytes, with CD14^+^ classical monocytes predominating). The results showed that the ANNEXIN pathway was significantly enhanced in CD8^+^ T cells (Figure 6F), plasma cells (Figure 6H), and proliferating cells (Figure 6K) (*p* < 0.01); the PECAM1 pathway was significantly enhanced in plasma cells (Figure 6I) and proliferating cells (Figure 6L) (*p* < 0.01) but significantly reduced in monocytes (Figure 6M) (*p* < 0.05); the CCL pathway was only marginally significantly enhanced in plasma cells (Figure 6G, *p* < 0.05); and the RESISTIN pathway was significantly enhanced only in proliferating cells (Figure 6J, *p* < 0.001). For the CysLTs, Cholesterol, and BAFF pathways, the number of detectable communication events per cell type was insufficient to permit reliable box plot comparisons. These results indicate that the communication enhancement of inflammatory pathways in BD is cell-subset-specific. The ANNEXIN pathway was broadly upregulated across multiple immune cell types, whereas CCL and RESISTIN were confined to plasma cells and proliferating cells, respectively. Notably, the communication probability of the PECAM1 pathway in monocytes was significantly reduced in BD (Figure 6M), consistent with their overall communication decline (Figure 6E) and the monocyte-focused network analysis (Figures 7A–C), further substantiating the bidirectional communication suppression of monocytes in BD. It should be noted that the “Monocyte” population in the communication analysis represents merged monocytes (predominantly CD14^+^ classical monocytes); thus, the communication suppression of this population primarily reflects the functional characteristics of classical monocytes, and the CCL pathway did not reach statistical significance (*p* = 0.116), with no apparent compensatory upregulation of pro-inflammatory pathways.

### 3.8 External validation using independent transcriptomic datasets

**Figure 8:**
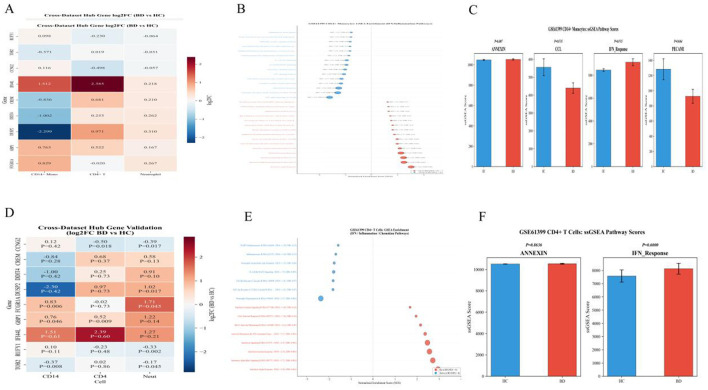
External validation of LASSE-selected hub genes and interferon signaling activation. **(A)** Heatmap showing expression of nine LASSO-selected hub genes across three independent datasets: GSE61399_CD14+ monocytes, GSE61399_CD4+ T cells, and GSE205867_neutrophils. Red indicates higher expression in BD. All nine genes were consistently upregulated in CD14+ monocytes. **(B)** GSEA confirms strong activation of type I interferon signaling in CD14+ monocytes, with significant enrichment of Interferon Alpha Response (NES = +3.04, FDR < 0.001) and Interferon Gamma Response (NES = +2.26, FDR < 0.001). **(C)** ssGSEA pathway scoring further confirms IFN_Response activation in CD14+ monocytes (BD vs. HC, *p* = 0.075). **(D)** Heatmap of log2FC values for the nine hub genes across the three datasets. Red indicates upregulation in BD; blue indicates downregulation. **(E)** GSEA reveals consistent activation of Interferon Alpha Response (NES = +2.96, FDR < 0.001) and Interferon Gamma Response (NES = +2.56, FDR < 0.001) in CD4+ T cells. **(F)** ssGSEA pathway scoring shows a non-significant elevation of IFN_Response in CD4+ T cells (BD vs. HC, *p* = 0.600, ns). BD, Behçet's disease; FDR, false discovery rate; GSEA, gene set enrichment analysis; HC, healthy control; NES, normalized enrichment score; ns, not significant; ssGSEA, single-sample gene set enrichment analysis.

To evaluate the generalizability of our findings beyond the discovery cohort, we performed validation analyses using two independent transcriptomic datasets: GSE61399 (purified CD14^+^ monocytes and CD4^+^ T cells) and GSE205867 (peripheral blood neutrophils). In CD14^+^ monocytes, all nine LASSO-selected core genes showed consistent upregulation in the BD group relative to the HC group (Figures 8A, D). GSEA confirmed robust activation of type I interferon signaling in CD14^+^ monocytes, with both interferon alpha response (NES = +3.04, FDR < 0.001) and interferon gamma response (NES = +2.26, FDR < 0.001) significantly enriched (Figure 8B). ssGSEA pathway scoring further revealed a trend toward IFN_Response activation in CD14^+^ monocytes (*p* = 0.075, Figure 8C). In CD4^+^ T cells, GSEA likewise showed strong enrichment of interferon alpha response (NES = +2.96, FDR < 0.001) and interferon gamma response (NES = +2.56, FDR < 0.001) (Figure 8E), and ssGSEA indicated a non-significant upward trend in IFN_Response scores in BD (Figure 8F; *p* = 0.600). These cross-platform, cross-cell-type validations—spanning microarray and RNA-seq technologies and three distinct immune lineages (monocytes, T cells, and neutrophils)—robustly support the key findings of our study, particularly the activation of interferon signaling in BD. Notably, monocytes displayed overall communication suppression yet exhibited strong transcriptional activation of interferon signaling and LASSO core genes, suggesting a subtype-specific regulatory state of “transcriptionally active yet communication-suppressed.”

## 4 Conclusions

This study reveals the remodeling features of the peripheral blood immune landscape in Behçet's disease (BD) through single-cell transcriptomics. BD patients exhibited subset-specific monocyte redistribution at the group level, with CD14^+^ classical monocytes showing a significantly higher proportion (8.54% vs. 1.51%, *p* < 0.001) and FCGR3A^+^ non-classical monocytes markedly reduced (3.04% vs. 7.23%, *p* < 0.001), accompanied by elevated proportions of CD8^+^ T cells, CD4^+^ T cells, plasma cells, and proliferating cells. Differential expression analysis identified 86 upregulated and 84 downregulated genes, primarily enriched in interferon/inflammatory pathways. Among the nine feature genes selected by LASSO regression, IFI44L demonstrated moderate diagnostic potential (AUC = 0.734). Intercellular communication analysis further revealed attenuated communication in costimulatory pathways such as CD40 and ICAM in BD, whereas the ANNEXIN pathway was significantly enhanced in CD8^+^ T cells, plasma cells, and proliferating cells, and the PECAM1 pathway was increased in plasma cells and proliferating cells but significantly reduced in monocytes. Notably, monocytes exhibited overall communication suppression and PECAM1 pathway downregulation at the communication level, contrasting with the strong activation of interferon signaling and LASSO core genes observed in external validation, forming a decoupling phenomenon of “transcriptionally active yet communication-suppressed.” This finding offers a new perspective for understanding subtype-specific immune regulation and the mechanisms of persistent inflammation in BD, and provides a theoretical basis for diagnostic biomarker development and communication-targeted therapeutic strategies.

## 5 Discussion

In this study, we employed single-cell transcriptomics to systematically delineate the peripheral blood immune cell landscape and intercellular communication network remodeling in Behçet's disease. Our analysis revealed subset-specific monocyte redistribution, characterized by a marked reduction in FCGR3A^+^ non-classical monocytes and an increased proportion of CD14^+^ classical monocytes at the group level. Per-subject analyses (Supplementary Figure S1B) showed that these directional trends were consistently observed in the majority of BD patients, although individual-level statistical significance was not reached, likely due to the limited sample size (*n* = 8 per group), alongside elevated proportions of CD8^+^ T cells, CD4^+^ T cells, plasma cells, and proliferating cells. Individual-level UMAP projections confirmed that the observed clustering patterns and cell-type distributions were broadly consistent across donors within each group, supporting the robustness of our group-level comparisons. The observed decrease in non-classical monocytes reflects a reduction in their relative abundance within the PBMC pool rather than an absolute decline in peripheral blood monocyte counts. This relative decrease may be attributable, at least in part, to the proportional increase of T cell subsets, which enlarges the total immune cell pool and thereby reduces the fractional contribution of non-classical monocytes.

Differentially expressed genes were enriched in interferon and inflammatory pathways. LASSO regression identified nine feature genes, among which IFI44L showed moderate diagnostic potential (AUC = 0.734) but did not reach statistical significance (*p* = 0.1304), likely owing to insufficient statistical power from the small discovery cohort. Intercellular communication analysis further revealed attenuated costimulatory signaling (e.g., CD40, ICAM) in BD, whereas inflammatory/metabolic pathways, including CCL, CysLTs, RESISTIN, ANNEXIN, PECAM1, Cholesterol, and BAFF—showed relatively enhanced communication intensity at the overall level. At the subset level, the ANNEXIN pathway was significantly enhanced in CD8^+^ T cells, plasma cells, and proliferating cells; the PECAM1 pathway was enhanced in plasma cells and proliferating cells but significantly reduced in monocytes; the CCL pathway was only marginally significantly enhanced in plasma cells; and the RESISTIN pathway was significantly enhanced only in proliferating cells. Notably, despite the overall decline in monocyte communication, monocytes still exhibited strong transcriptional activation of interferon signaling and LASSO core genes, pointing to a decoupling phenomenon of “transcriptionally active yet communication-suppressed.”

It should be clarified that the “Monocyte” population in the communication analysis was treated as a merged group of three monocyte subsets (CD14^+^ classical, FCGR3A^+^ non-classical, and FCN1^+^ myeloid progenitors) rather than analyzing CD14^+^ classical monocytes separately, based on the following considerations. First, CellChat's communication probability calculation depends on an adequate number of cells per population to ensure statistical robustness. Non-classical monocytes in the BD group were extremely scarce (only 3.04% of PBMCs, approximately 1,358 cells); splitting them from classical monocytes would render communication events for this subset severely sparse due to insufficient sample size, precluding reliable detection of numerous ligand–receptor pairs. Moreover, classical monocytes in the HC group were likewise limited (1.51%, approximately 614 cells), such that separate analysis would compromise the fairness of between-group communication comparisons. Second, proportion analysis confirmed that CD14^+^ classical monocytes account for over 52% of total monocytes, constituting the overwhelmingly dominant subset; the communication characteristics of the merged population are therefore inherently governed by this subset. Thus, merged analysis both preserves statistical power and reasonably reflects the communication changes of classical monocytes. This strategy has been widely adopted in published single-cell communication studies. Furthermore, we separately present the abundance differences of the three subsets through proportion analysis (Supplementary Figure S2), providing reviewers and readers with subset-level evidence of change. Resolving the communication characteristics of non-classical monocytes will require accumulating sufficient cell numbers in larger independent cohorts.

Cross-platform validation using two independent transcriptomic datasets (GSE61399 and GSE205867) further supported these findings. All nine LASSO-selected core genes showed consistent upregulation in CD14^+^ monocytes, and type I interferon signaling was robustly enriched in both CD14^+^ monocytes and CD4^+^ T cells (Figures 8A–F). Given that this is a discovery-driven computational study based on public datasets, our findings should be regarded as hypothesis-generating rather than definitive conclusions. The rarity of BD and the treatment-related confounders inherent in patient samples underscore the necessity of supplementary validation through independent datasets and future experimental studies.

The subset-specific monocyte redistribution identified in this study has important implications for interpreting our CellChat communication analysis. The merged “Monocyte” population in CellChat was treated as a unified group comprising three subsets (CD14^+^ classical, FCGR3A^+^ non-classical, and FCN1^+^ myeloid progenitors). This merged population is numerically dominated by CD14^+^ classical monocytes, which constitute the majority of monocytes in BD. The preferential loss of FCGR3A^+^ non-classical monocytes—known to play important roles in immune surveillance and vascular homeostasis—likely contributes to the reduced global communication observed for the monocyte population as a whole. Conversely, the increased proportion of CD14^+^ classical monocytes, which are more pro-inflammatory in nature, may partially underpin the transcriptional activation of interferon signaling and LASSO-selected core genes observed in external validation. We interpret this as a “proportion-driven functional shift” rather than “functional compensation” *per se*, as the merged monocyte population predominantly reflects the characteristics of the classical subset. Resolving the communication characteristics of non-classical monocytes will require accumulation of sufficient cell numbers in larger independent cohorts.

Several aspects of this work merit attention. Delineating the peripheral blood immune landscape of BD at single-cell resolution helps fill gaps in our understanding of cellular heterogeneity. To our knowledge, this is also the first study to systematically compare intercellular communication across seven inflammatory and metabolism-related pathways in BD, revealing subset-specific signaling enhancement. The coexistence of monocyte proportional increase and communication suppression—the “transcriptionally active yet communication-suppressed” decoupling phenomenon—offers a novel hypothesis for persistent inflammation. Moreover, the nine-gene signature, particularly IFI44L, demonstrated moderate diagnostic potential, although evaluation in larger independent cohorts is required before clinical application. Finally, the subset-specific activation of the ANNEXIN, PECAM1, CCL, and RESISTIN pathways provides transcriptomic support for the four-stage inflammatory cascade hypothesis outlined in our Introduction.

Several limitations should be acknowledged. The small sample size (n = 8 patients per group) limited statistical power and increased the risk of LASSO model overfitting. Although 10-fold cross-validation was employed to mitigate overfitting, the LASSO results should still be considered preliminary, and the nine-gene signature requires validation in larger independent cohorts—a challenge commonly encountered in rare disease research. Furthermore, the GSE198616 dataset lacks detailed clinical annotations, including disease subtype (mucocutaneous, ocular, vascular, or neurological), disease activity scores (e.g., BDCAF), and treatment status. These factors may substantially influence transcriptomic profiles and immune cell composition. Some studies have reported peripheral blood monocytosis in BD patients (21), and BD can be classified into subtypes such as neurological, gastrointestinal, and vascular; however, this dataset does not specify which subtype each patient belongs to (22). More refined subgroup analyses are needed to accurately delineate the peripheral blood immune landscape across different BD subtypes.

The intercellular communication analysis was performed using CellChat, which predicts communication probabilities based on ligand–receptor co-expression within transcriptomic data. These predictions reflect transcriptional potential rather than direct measurements of intercellular communication or functional output. Consequently, our findings of enhanced pathway communication should be interpreted as selective transcriptional enrichment rather than definitive evidence of functional compensation. Comparisons of pathways such as CysLTs, Cholesterol, and BAFF were further constrained by data sparsity, precluding full visualization of their communication differences. Future studies integrating spatial transcriptomics, functional assays, or proteomic approaches will be needed to verify whether these transcriptional signals translate into functional intercellular communication.

The lack of experimental validation (e.g., qPCR, flow cytometry, or ELISA) is another noteworthy limitation. Although cross-platform validation using two independent transcriptomic datasets provided supportive evidence, transcriptomic data cannot substitute for protein-level or functional confirmation. Experimental validation in BD faces two major challenges: the rarity of the disease makes it difficult to assemble independent cohorts with adequate power, and most patients receive immunomodulatory therapy, which significantly confounds transcriptomic and immunological measurements. These challenges are widely recognized within the BD research community and are frequently cited as limiting factors in biomarker validation studies (13–15). Future multicenter collaborative efforts will be essential to overcome these barriers. Finally, although single-cell differential expression analysis may be subject to pseudoreplication due to the hierarchical nature of the data (cells nested within individuals), our key findings have been validated using independent bulk datasets with true biological replicates; the DEG results should therefore be considered hypothesis-generating rather than confirmatory.

We acknowledge that our primary differential expression analysis was performed at the single-cell level, which may be subject to pseudoreplication. To address this, we performed an additional pseudobulk analysis aggregating cells by donor. The core genes IFI44L, GBP1, and FCGR1A showed consistent upregulation trends in BD, further supporting the robustness of our findings, although statistical significance was attenuated due to the limited sample size. These results are presented in Supplementary Table S4.

Despite these limitations, this study provides a single-cell perspective on the communication remodeling between multiple peripheral blood immune subsets and signaling pathways in BD, an area that has not been systematically investigated. These results may help guide more precise therapeutic strategies. Going forward, independent peripheral blood cohorts will be needed to confirm monocyte subset alterations via flow cytometry and to measure feature genes such as IFI44L by qRT-PCR or western blotting. Larger single-cell datasets, particularly those encompassing samples across different disease activity levels and clinical subtypes, will be critical for resolving subset-specific communication differences and elucidating how BD develops.

## Data Availability

The datasets presented in this study can be found in online repositories. The names of the repository/repositories and accession number(s) can be found in the article/Supplementary material.
