# Supplementary material for: Redistribution of peripheral blood immune cell subsets and remodeling of intercellular communication in rare Behçet's disease: a single-cell transcriptomics study
Source: Front Med (Lausanne). 2026 Aug 4;13:1908366. doi: 10.3389/fmed.2026.1908366 (PMC13481179; doi:10.3389/fmed.2026.1908366)
Supplement: Supplementary file 1 [file Supplementary_File_1.pdf]

Supplementary Figure S1. Detailed monocyte subset composition in BD and HC.

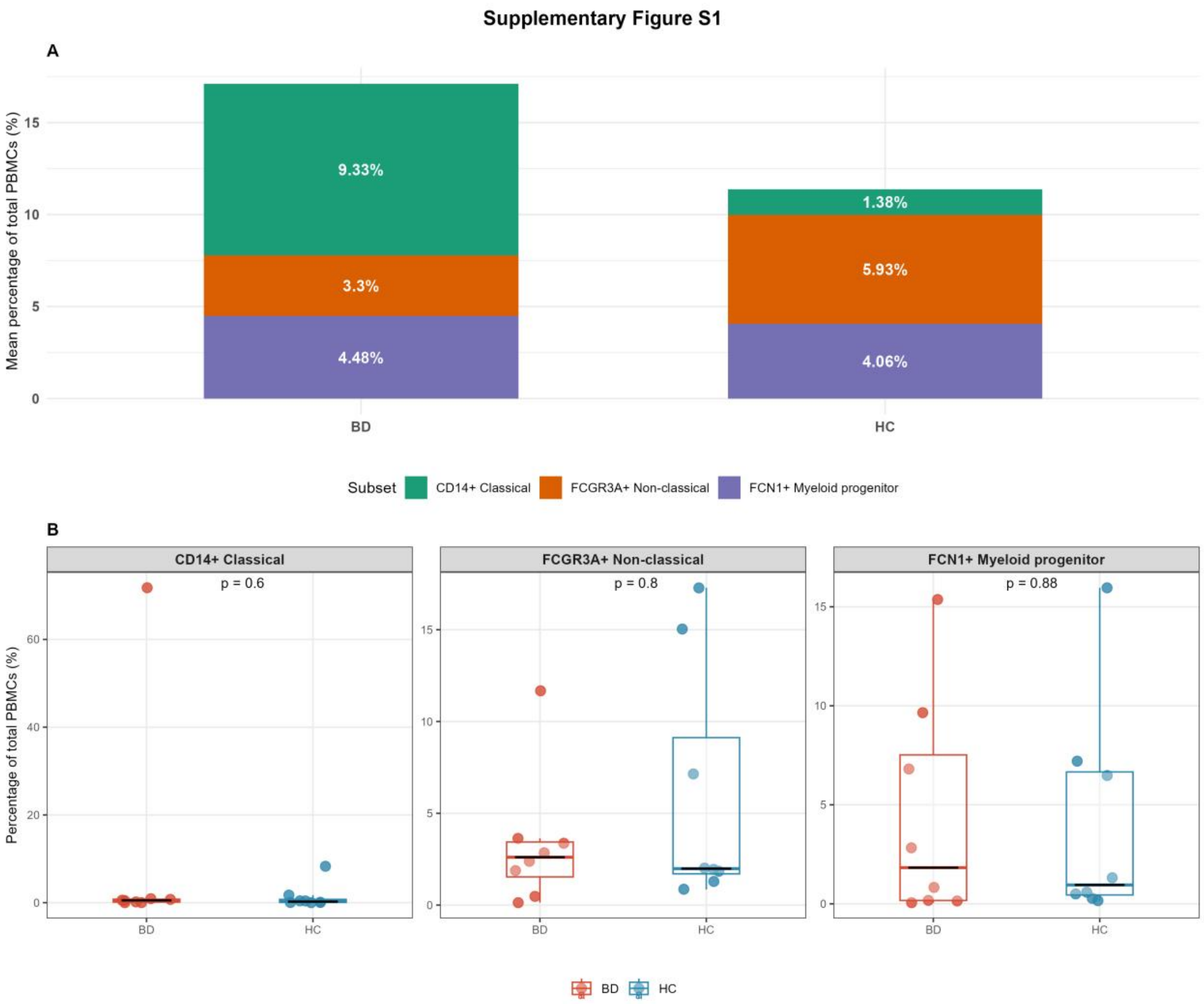

(A) Stacked bar plot showing group-level proportions of CD14$^+$ classical monocytes (Cluster 7), FCGR3A$^+$ non-classical monocytes (Cluster 9), and FCN1$^+$ myeloid progenitors (Cluster 11) as percentages of total PBMCs in BD and HC groups. Numerical values are displayed on each bar segment. CD14$^+$ classical monocytes showed a significantly higher proportion in BD (8.54% vs 1.51%, $p<0.001$), whereas FCGR3A$^+$ non-classical monocytes were markedly reduced (3.04% vs 7.23%, $p<0.001$). FCN1$^+$ myeloid progenitors showed a modest, non-significant increase in BD (4.76% vs 4.19%, ns). (B) Per-subject scatter plot showing the proportion of each monocyte subset relative to total PBMCs for individual BD and HC donors. Each dot represents one donor; horizontal lines indicate group medians. Directional trends—higher CD14$^+$ classical monocyte proportions and lower FCGR3A$^+$ non-classical monocyte proportions in BD—were consistently observed in the majority of BD patients, despite limited statistical power at the individual level due to the small sample size (n=8 per group). These subset-specific patterns contrast with the overall monocyte proportion shown in Figure 1D, highlighting that the reduced monocyte abundance observed at the bulk level is primarily driven by loss of the FCGR3A$^+$ non-classical subset.

Supplementary Figure S2. Comparison of overall communication probabilities for four representative signaling pathways between BD and HC.

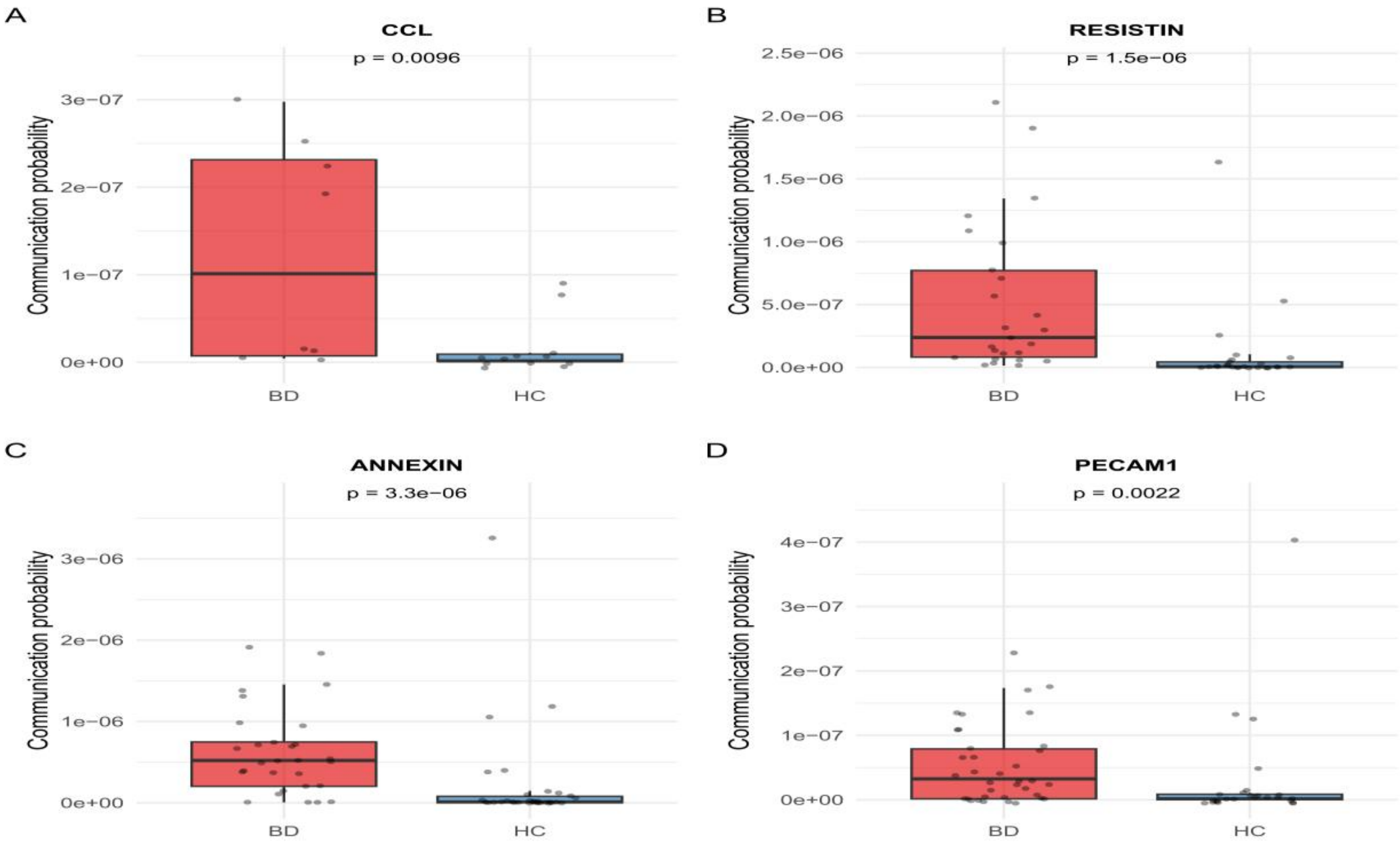

(A–D) Boxplots showing the overall communication probabilities of CCL (A), RESISTIN (B), ANNEXIN (C), and PECAM1 (D) pathways in BD (red) versus HC (blue). Each dot represents a ligand-receptor pair involving any cell type. All four pathways showed slightly higher communication probabilities in BD, consistent with the differential pathway screening results shown in Figure 5A. P values were calculated using two-sided Wilcoxon rank-sum test and are indicated on each panel. Abbreviations: BD, Behçet's disease; HC, healthy control.

Supplementary Figure S3. UMAP projections of each individual sample colored by cluster.

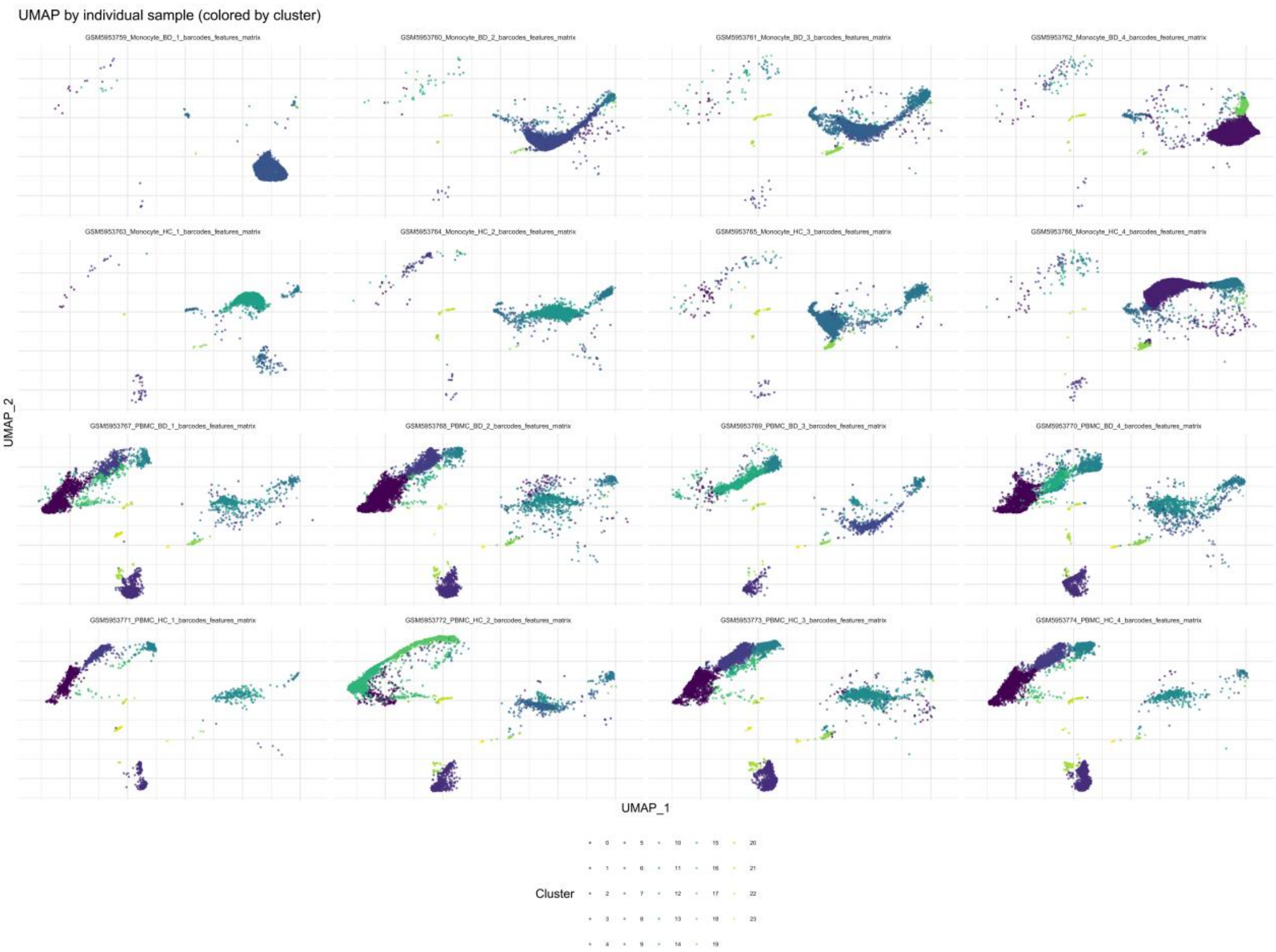

UMAP projections of all 16 samples (8 BD patients and 8 healthy controls), with each panel representing one individual donor. Colors indicate cluster identity as defined in Figure 1B. The overall clustering patterns are broadly consistent across individuals within each group, confirming that the observed group-level differences are not driven by any single donor.
