# Supplementary material for: Redistribution of peripheral blood immune cell subsets and remodeling of intercellular communication in rare Behçet's disease: a single-cell transcriptomics study
Source: Front Med (Lausanne). 2026 Aug 4;13:1908366. doi: 10.3389/fmed.2026.1908366 (PMC13481179; doi:10.3389/fmed.2026.1908366)
Supplement: Supplementary file 2 [file Table_1.docx]

| name | contribut contribut group | contribut name  diff abs_  HC  BD |
| --- | --- | --- |
| MHC-I | 6.477726270.10368543HC | <dbl> <dbl> <dbl>  2  <fct> |
| MIF | 5.598728070.1021410 BD | 0.9 1 CD40 0 0.0674 -0.0674 |
| MHC-II | 5.36511420.10169828HC | 1 2 APP 0 0.0640 -0.0640 |
| MIF | 4.9294459 0.10082984HC | 0.9 3 SN 0.0586 0  0.0586 |
| MHC-I | 4.17664180.09917272BD | 2 4 SEMA4 0 0.0585 -0.0585 |
| MHC-II | 3.625720880.09780073BD | 1 5 VISFATIN 0 0.0511 -0.0511 |
| CD99 | 1.958986050.09224668HC | 3 6 CysLTs 0.0664 0.0397  0.0267 |
| GALECTIN | 1.956589760.09223626BD | 1 7 ICAM 0.0684 0.0805 -0.0121 |
| GALECTIN | 1.91972980.09207474HC | 1 8 RESISTIN 0.0889 0.0782  0.0107 |
| ANNEXIN | 1.816228330.09160727BD | 0.4 9 CD23 0.0623 0.0722 -0.00995 |
| RESISTIN | 1.298132940.08887310BD | 0.0724 0.0647 0.00763  0.2 10  CCL |
| CypA | 1.193412940.08821369HC | 0.0849 0.0922 -0.00736  2 11  CD99 |
| CD99 | 7.650107090.08488394BD | 0.0916 0.0843 0.00735  3 12  ANNEXIN |
| CypA | 7.301685180.08454939BD | 0.0592 0.0650 -0.00576  2 13  LCK |
| ANNEXIN | 7.0110653 0.08426004HC | 0.0611 0.0662 -0.00512  IL16  0.4 14 |
| ICAM | 4.028332320.0805013 HC | 0.0740 0.0789 -0.00489  9 15  CLEC |
| SELPLG | 3.833722320.08018169HC | 0.0759 0.0710 0.00487  2 16  PECAM1 |
| CLEC | 3.124392520.07888755HC | 0.0992 0.104  2 17  MHC-I  -0.00451 |
| RESISTIN | 2.804906980.07822192HC | 0.0637 0.0593 0.00436  0.2 18  Cholesterol |
| SELPLG | 1.998760150.07620218BD | 0.0724 0.0681 0.00432 0 0762 0 0802 -0 00398  2 19  20  BAFF  SELPLG |

_diff

<dbl>

0.0674 0.0640 0.0586 0.0585 0.0511 0.0267 0.0121 0.0107 0.00995

0.00763 0.00736 0.00735 0.00576 0.00512 0.00489 0.00487 0.00451 0.00436 0.00432 0.00398
