# Supplementary material for: Redistribution of peripheral blood immune cell subsets and remodeling of intercellular communication in rare Behçet's disease: a single-cell transcriptomics study
Source: Front Med (Lausanne). 2026 Aug 4;13:1908366. doi: 10.3389/fmed.2026.1908366 (PMC13481179; doi:10.3389/fmed.2026.1908366)
Supplement: Supplementary file 3 [file Table_2.docx]

name	contribut contribut group	contribution.relative.1

MIF	5.598728070.1021410 BD	0.9

MHC-I	4.17664180.09917272BD	2

MHC-II	3.625720880.09780073BD	1

GALECTIN 1.956589760.09223626BD	1

ANNEXIN 1.816228330.09160727BD	0.4

RESISTIN 1.298132940.08887310BD	0.2

CD99	7.650107090.08488394BD	3

CypA	7.301685180.08454939BD	2

SELPLG	1.998760150.07620218BD	2

PECAM1	1.9021249 0.07591550BD	0.4

CLEC	1.352720260.07400066BD	2

BAFF	1.011035230.07243995BD	0.4

CCL	9.9710779 0.07236724BD	0.2

ADGRE	9.395067690.07205695BD	1

ICAM	4.46785750.06839390BD	9

CD45	4.110787550.06800648BD	2

CysLTs	2.879860230.06639950BD	4e-05

Cholestero1.517321050.06368960BD	0.3

CD23	1.065298870.0622865 BD	9

IL16	7.795777880.06109818BD	4
