# Supplementary material for: Redistribution of peripheral blood immune cell subsets and remodeling of intercellular communication in rare Behçet's disease: a single-cell transcriptomics study
Source: Front Med (Lausanne). 2026 Aug 4;13:1908366. doi: 10.3389/fmed.2026.1908366 (PMC13481179; doi:10.3389/fmed.2026.1908366)
Supplement: Supplementary file 4 [file Table_3.docx]

## **Supplementary Table S3. Leave-one-out sensitivity analysis of IFI44L expression**

| Removed BD sample | log2FC (BD vs HC) | P-value |
| --- | --- | --- |
| 1 | 0.41 | 0.232 |
| 2 | 1.32 | 0.189 |
| 3 | 1.32 | 0.189 |
| 4 | 1.33 | 0.189 |
| 5 | 1.45 | 0.029 |
| 6 | 1.39 | 0.189 |
| 7 | 1.41 | 0.152 |
| 8 | 1.41 | 0.121 |

log2FC remained positive across all iterations, confirming that the upregulation trend is not attributable to any single sample.
