# Supplementary material for: Redistribution of peripheral blood immune cell subsets and remodeling of intercellular communication in rare Behçet's disease: a single-cell transcriptomics study
Source: Front Med (Lausanne). 2026 Aug 4;13:1908366. doi: 10.3389/fmed.2026.1908366 (PMC13481179; doi:10.3389/fmed.2026.1908366)
Supplement: Supplementary file 5 [file Table_4.doc]

**Supplementary Table S4：pseudobulk_core_genes_results**

| Gene | log2FoldChange | pvalue | padj | Trend in BD |
| --- | --- | --- | --- | --- |
| IFI44L | +1.55 | 0.004 | 0.380 | Up |
| GBP1 | +1.29 | 0.010 | 0.545 | Up |
| FCGR1A | +1.45 | 0.047 | 0.814 | Up |
| CREM | +2.21 | 0.0005 | 0.175 | Up |
| DDIT4 | +1.65 | 0.013 | 0.577 | Up |
| DUSP2 | +1.39 | 0.029 | 0.707 | Up |
| RUFY1 | -0.33 | 0.007 | 0.475 | Down |
| CCNG2 | -0.48 | 0.038 | 0.774 | Down |
| TOB2 | -0.82 | 0.00002 | 0.034 | Down |

Pseudobulk differential expression analysis was performed using DESeq2 with cells aggregated by donor (n=8 BD vs 8 HC). Positive log2FoldChange indicates upregulation in BD. Core genes IFI44L, GBP1, and FCGR1A showed consistent upregulation trends, supporting the robustness of our single-cell findings at the individual level. Statistical significance was limited by the small sample size.
